# Protective Effects of a Lipid Extract from Hard-Shelled Mussel (*Mytilus coruscus*) on Intestinal Integrity after Lipopolysaccharide Challenge in Mice

**DOI:** 10.3390/nu10070860

**Published:** 2018-07-03

**Authors:** Yi Wan, Yuanqing Fu, Fenglei Wang, Andrew J. Sinclair, Duo Li

**Affiliations:** 1 Institution of Nutrition and Health, Qingdao University, Qingdao 266071, China; yiwanzju@163.com; 2 Department of Food Science and Nutrition, Zhejiang University, Hangzhou 310058, China; fuyuanqing@163.com (Y.F.); fengleiwang@zju.edu.cn (F.W.); 3 Department of Maternal and Infant Nutrition Research, Beingmate Baby and Child Food Co., Ltd., Hangzhou 311106, China; 4 School of Medicine, Deakin University, Locked Bag, Geelong 20000, Australia; andrew.sinclair@deakin.edu.au

**Keywords:** hard-shelled mussel, *Mytilus coruscus*, intestinal integrity, lipopolysaccharide, TLR-4 signaling pathway

## Abstract

This study investigated the protective effects of a lipid extract from hard-shelled mussel (HMLE) on intestinal integrity and the underlying mechanisms after a lipopolysaccharide (LPS) challenge in mice by using a 3 × 2 factorial design. Mice received olive oil, fish oil, and HMLE (*n* = 12 per group) by using gastric gavage for six weeks, respectively. Then half the mice in each group was injected intraperitoneally with LPS and the other half with phosphate buffered saline. Four hours after injection, mice were sacrificed and samples were collected. n-3 PUFAs were significantly enriched in erythrocytes following fish oil and HMLE supplementation. Both fish oil and HMLE improved intestinal morphology by restoring the ileac villus height and barrier function, which is indicated by decreased colonic myeloperoxidase activity and increased diamine oxidase activity as well as enhanced mRNA expression of intestinal tight junction proteins known as occludin and claudin-1 when compared with olive oil. In addition, both fish oil and HMLE increased colon production and the expression of anti-inflammatory cytokine, IL-10, while they inhibited the abnormal production and expression of pro-inflammatory cytokines including TNF-α, IL-1β, and IL-6 relative to the olive oil. Lastly, in comparison with olive oil, both fish oil and HMLE downregulated the TLR-4 signaling pathway by reducing the expression of two key molecules in this pathway, which are called TLR-4 and MyD88. These results suggest that HMLE had a protective effect on intestinal integrity after the LPS challenge, which was equivalent to that of fish oil. This effect might be associated with the regulation of inflammatory mediators and the inhibition of the TLR-4 signaling pathway.

## 1. Introduction

Intestinal integrity is very important since it constitutes an essential barrier to protect against the diffusion of pathogens, toxins, and allergens from the external environment [1,2]. However, many factors such as infection and inflammation can induce intestinal injury especially disruption of the intestinal barrier [3]. A dysfunctional intestinal barrier is associated with the pathophysiology of various diseases and disorders such as inflammatory bowel disease, irritable bowel syndrome, and celiac disease [1]. It has been widely accepted that over-release of pro-inflammatory cytokines play a crucial role in intestinal barrier disturbance through different mechanisms including induction of gross lesions, reduction in tight junction strands, upregulation of epithelial apoptosis, and arrested epithelial restitution [4,5]. Therefore, anti-inflammatory treatment aimed to inhibit the production of intestinal pro-inflammatory cytokines may have beneficial effects in alleviating intestinal injury.

The hard-shelled mussel (*Mytilus coruscus*) is the main mussel species commonly cultivated in coastal areas of China and is the most representative mollusk in the Chinese bivalve market [6]. Our previous study examining its nutrient composition showed that a hard-shelled mussel is rich in n-3 polyunsaturated fatty acids (PUFA) ranging from 34% to 37% of total fatty acids [7]. Eicosapentaenoic acid (EPA, 11–15% of total fatty acids) and docosahexaenoic acid (DHA, 12–18%) are the most abundant n-3 PUFA. Long chain n-3 PUFA such as EPA and DHA are generally believed to have anti-inflammatory effects [8,9,10] and have been used as immune modulators [11,12]. Therefore, EPA-rich and DHA-rich hard shelled mussel could be used as part of an anti-inflammatory treatment. In recent years, both animal and human studies have demonstrated that the lipid extract from hard-shelled mussels (hard-shelled mussel lipid extract, HMLE) exert strong anti-inflammatory effects on arthritis and inflammatory bowel disease [6,13,14,15]. Further examination showed that HMLE could downregulate pro-inflammatory cytokines including the tumor necrosis factor (TNF)-α, interleukin (IL)-1β and IL-6, and upregulated anti-inflammatory cytokines including IL-4 and IL-10, which indicates that the strong activity may be ascribed to the regulation of balance between pro-inflammatory and anti-inflammatory cytokines [15]. However, there is no report on the anti-inflammatory effects of HMLE on intestinal injury and the underlying molecular mechanism(s).

In this study, we aimed to investigate the potential protective effects of HMLE on intestinal integrity after a lipopolysaccharide (LPS) challenge in mice. We performed intestinal morphology examination and measured the colon tissue concentration of myeloperoxidase (MPO), which is an inflammation marker whose concentration increases and undergoes inflammation, and diamine oxidase (DAO), which is a mucosal injury marker that decreases when mucosal cells are injured, and cytokines such as IL-1β, IL-6, IL-10, and TNF-α. In addition, we determined the mRNA expression of tight junction proteins including occludin and claudin-1, which are major determinants of an intestinal barrier function [16]. In order to explore the intracellular signaling mechanism underlying the potential effect of HMLE, we also measured the mRNA expression of cytokines and key molecules in a toll-like receptor (TLR)-4 signaling pathway, which is a pathway related to the intestinal mucosa barrier function [17]. These molecules include TLR-4, myeloid differentitation factor 88 (MyD88), IL-1 receptor-associated kinase (IRAK)-4, and TNF receptor-associated factor (TRAF)-6.

## 2. Materials and Methods

### 2.1. Chemicals and Reagents

Olive oil was purchased from a local supermarket in Hangzhou. Fish oil was purchased from Neptunus Bioengineering Co., Ltd., (Hangzhou, China). LPS was obtained from Sigma-Aldrich (St. Louis, MO, USA). Commercial kits for determining myeloperoxidase (MPO) and diamine oxidase (DAO) were purchased from Nanjing Jiancheng Bioengineering Institute (Nanjing, China). A total protein determination kit (BCA Protein Assay Kit) was acquired from Beyotime Institute of Biotechnology (Haimen, China). Cytokines measurement kits were purchased from Lianke Biotechnology CO., Ltd., (Hangzhou, China). RNAiso Plus Kit, Prime Script RT Reagent Kit, and SYBR Premix Ex Taq TM (Tli RNaseH Plus) Kit were purchased from Takara Biotechnology (Dalian) Co., Ltd., (Dalian, China). Sulphuric acid, methanol, chloroform, acetic acid, diethyl ether, and petroleum ether were purchased from the Sinopharm Chemical Reagent Co., Ltd., (Shanghai, China) and were of an analytical grade. N-hexane was purchased from the Tianjin Shield Specialty Chemical Co., Ltd., (Tianjin, China) and was of an HPLC grade.

### 2.2. Preparation of Hard-Shelled Mussel Lipid Extract (HMLE)

Hard-shelled mussels were collected from the Shengsi Islands in the Zhejiang province and HMLE was prepared through the procedure of CO_2_ supercritical fluid extraction, which was previously described [6]. Lipid composition of HMLE was also measured and reported in our previous study [7]. The lipid extract was then stored at −80 °C in amber vials to minimize autoxidation before use. 

### 2.3. Animals and Experimental Design

Thirty-six 6-week-old male C57 mice were obtained from the Laboratory Animal Center of Zhejiang University. They were maintained in an air-conditioned specific pathogen-free room with a controlled temperature (22 ± 1 °C), constant humidity (55 ± 5%), and a normal 12-h light/dark cycle. The animals were allowed *ad libitum* access to water and standard chows provided by the Laboratory Animal Center during the experimental period. All experimental procedures were approved by the Ethics Committee of the College of Biosystem Engineering & Food Science at Zhejiang University (Reference: 2014005).

The experiment was performed as a 3 × 2 factorial design with the main factors being gavage treatment and the LPS challenge. After one week of adaption, mice were randomly assigned to 3 groups (*n* = 12 per group) including the solvent control group, the fish oil control group, and the HMLE group. The solvent control group received olive oil by gavage at a volume of 5 mL/kg body weight daily [18]. Olive oil was commonly chosen as the solvent for mussel lipid extracts [6,19,20]. Fish oil and HMLE groups received the same volume of olive oil containing fish oil and HMLE, respectively. The dose of HMLE and fish oil was 300 mg/kg (around 6–8 mg/mouse). This dose was adapted from a previous study where the New Zealand green-lipped mussel lipid extract (GMLE) was administrated to mice with inflammatory bowel disease at a dose of 5 mg/mouse. It concluded that the optimal dose might not have been achieved [20]. The fish oil group was selected as a positive control since it has already been shown to enhance intestinal integrity in weaned pigs after the LPS challenge [21]. The fatty acid profile of olive oil, fish oil, and HMLE used in this study is shown in Table 1. The calculated n-3 fatty acid dose of three groups are 26.1 mg/kg (olive oil as linolenic acid), 208.7 mg/kg (fish oil, mainly EPA and DHA), and 124.2 mg/kg (HMLE, mainly EPA and DHA).

**Figure 1:**
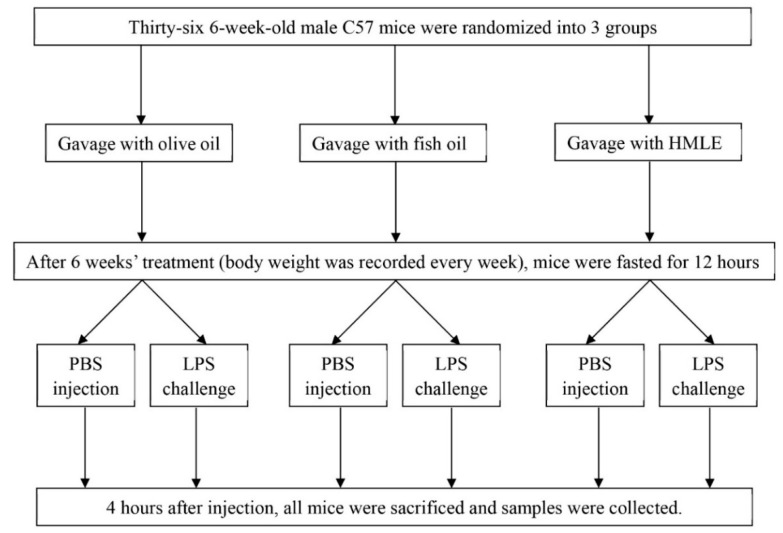
Schematic of study design.

During the treatment period, the body weight of mice was recorded at the same time of the day every week. After 6 weeks, all mice underwent fasting for 12 h based on a previous study [21]. Then each treatment group was further randomly divided into two subgroups in which one received the lipopolysaccharide (LPS) challenge and the other received sterile phosphate buffered saline (PBS). The LPS-challenged groups were injected intraperitoneally with LPS at a dose of 10 mg/kg body weight adapted from a previous study [22] while the unchallenged groups were injected with the same amount of PBS. Four hours after injection, all mice were sacrificed under anesthesia using diethyl ether. Blood samples were collected from the retro-orbital sinus to obtain erythrocytes. Ileac and colonic segments were excised, freed of adherent adipose tissue, and then flushed with ice-cold PBS. Afterward, ileac specimens were fixed in 10% formalin for morphology examination and colon samples were cut into small pieces (*n* = 4 in total) for further experiments, which is described below. The schematic of study design is shown in Figure 1.

### 2.4. Intestinal Morphology Examination

After a 24-h fixation in formalin, ileac segments were removed, dehydrated, and subsequently embedded in paraffin. Then the segments were cut in 5–8 μm thick sections. These samples were stained with hematoxylin and eosin and viewed under a light microscope (100×). The villus height and the associated crypt depth were measured in accordance with previous studies [23,24]. Villus height is defined as the distance from the villus tip to the crypt mouth and crypt depth is defined as the distance from the crypt mouth to the base.

### 2.5. Measurement of Erythrocyte Phospholipid Fatty Acids

Erythrocyte phospholipid fatty acids were measured using a standard method. The total lipids of erythrocytes were extracted with chloroform/methanol (1:1) and the phospholipid fraction was separated by using thin layer chromatography. Then the fatty acid methyl esters were prepared and determined by using gas-liquid chromatography, which was previously described [25].

### 2.6. MPO and DAO Activity Assay, and Cytokines Measurement

For MPO activity measurements, colon samples were weighed and homogenized in 20 volumes (*w*/*v*) of PBS containing 0.5% hexadecyltrimethylammonium. The homogenate was then centrifuged at 1480 g for 15 min and the resulting supernatant was assayed for MPO activity using detection kits. MPO activity was expressed in units per gram tissue. For DAO activity measurement, colon samples were weighed and immersed into 10 volumes (*w*/*v*) of saline and then centrifuged at 1480 g for 15 min. The supernatants were used to determine the total protein amount by utilizing the BCA Protein Assay Kit as well as to determine DAO activity using the assay kit. DAO activity was calculated and expressed in units per milligram protein. For colonic cytokines measurements, after centrifuging the 10% (*w*/*v*) colon homogenates at 1480 g for 15 min, the supernatant was directly used to determine cytokines including IL-1β, IL-6, IL-10, and TNF-α with the help of detection kits. All measurements were performed according to the manufacturers’ instructions.

### 2.7. Quantitative Real-Time Reverse Transcriptase-Polymerase Chain Reaction

Total RNA was isolated from colon samples using the RNAiso Plus Kit and cDNA was then synthesized using the Prime Script RT Reagent Kit. Quantitative real-time PCR was performed in the ABI ViiA 7 Real-Time PCR System (Applied Biosystems, Foster City, CA, USA) using the SYBR Premix Ex Taq TM (Tli RNaseH Plus) Kit, according to the manufacturers’ instructions. The following forward and reverse sequences were used: Occludin, forward 5′-GCTGTGATGTGTGTGAGCTG-3′ and reverse 5′-ATCTTTTGGGGTCCGTCAACT-3′; Claudin-1, forward 5′-TCAGGTCTGGCGACATTAGT-3′ and reverse 5′-GACAGGAGCAGGAAAGTAGGA-3′; IL-1β, forward 5′-GCAACTGTTCCTGAACTCAACT-3′ and reverse 5′-ATCTTTTGGGGTCCGTCAACT-3′; IL-6, forward 5′-CTTCCATCCAGTTGCCTTCTT-3′, and reverse 5′-AATTAAGCCTCCGACTTGTGAA-3′; IL-10, forward 5′-GCTCTTACTGACTGGCATGAG-3′ and reverse 5′-CGCAGCTCTAGGAGCATGTG-3′; TNF-α, forward 5′-CCCTCACACTCAGATCATCTTCT-3′, and reverse 5′-GCTACGACGTGGGCTACAG-3′; TLR-4, forward 5′-GGACTATGTGATGTGACCATTGAT-3′ and reverse 5′-TTATAGATACACCTGCCAGAGACA-3′; MyD88, forward 5′-CTACAGAGCAAGGAATGTGACT-3′ and reverse 5′-ACCTGATGCCATTTGCTGTCC-3′; IRAK4, forward 5′-ACATGCCCAACGGGTCCTT-3′ and reverse 5′-ACCTGATGCCATTTGCTGTCC-3′; TRAF6, forward 5′-ATCACTTGGCACGACACTTG-3′ and reverse 5′-TAGGCGACTCTCCAACTGTT-3′; and GAPDH, forward 5′-TGCTGAGTATGTCGTGGAGTC-3′ and reverse 5′-GGCGGAGATGATGACCCTT-3′. The thermal cycling conditions were 30s at 95 °C, which was followed by 40 cycles of denaturation at 95 °C for 5 s and annealing at 60 °C for 34 s. A melting curve analysis was performed at the end of the amplification cycles. The expression levels of target mRNA were normalized to GAPDH as an internal control and the relative quantification of gene expressions was performed using the 2^−ΔΔ^*C*_T_ method [26]. Results were expressed as relative expression ratios to the olive oil control group without the LPS challenge and the levels of gene expression in this group were set to 1.0 [27].

### 2.8. Statistical Analysis

The data were expressed as a mean ± standard deviation (SD) and analyzed with SPSS 23.0 statistical software. Differences in body weight and erythrocyte phospholipid fatty acids among three gavage treatments were tested using one-way ANOVA. This was followed by a Duncan’s post-hoc test. For main outcomes including intestinal morphology, activity of DAO and MPO, colonic cytokines concentrations, and mRNA expression levels, two-way ANOVA was conducted to study the interaction between gavage treatments and the LPS challenge. A significant interaction (*p* for interaction < 0.05) indicated that the effects of gavage treatments were different between the LPS challenged groups and the unchallenged groups. Interactions in which 0.05 < *p* < 0.10 were discussed as trends. When there was a significant treatment effect (*p* for LPS challenge < 0.05 or *p* for gavage treatment < 0.05). Afterward, a post-hoc test was conducted using one-way ANOVA followed by Duncan’s test to compare the group difference. Except where otherwise specified, a two-sided *p* value < 0.05 was considered significant. Superscripts were used to indicate the statistical differences and the letter ‘a’ represents the highest value.

## 3. Results

### 3.1. Change in the Body Weight and Erythrocyte Phospholipid Fatty Acids

**Figure 2:**
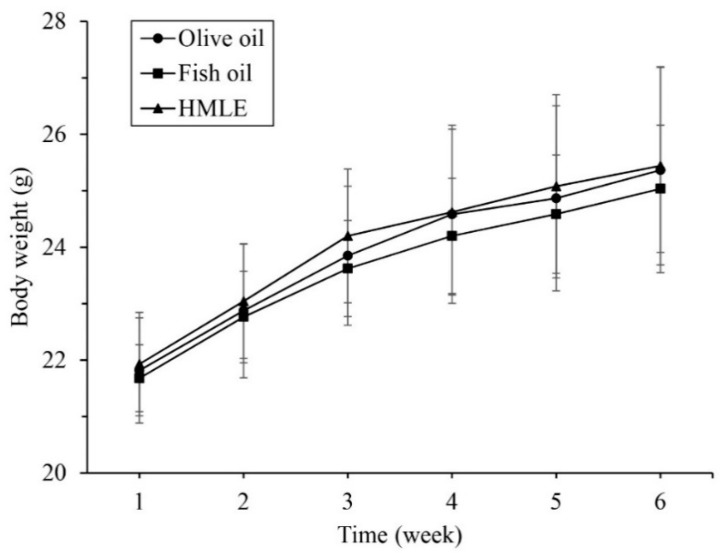
Changes in the body weight of mice. Data are represented as the mean ± SD.

The body weight of all the mice continued to increase with age during the six-week treatment (Figure 2). At each time point, there were no significant differences among olive oil control, fish oil control, and HMLE groups.

Erythrocyte phospholipid fatty acid composition after gavage treatment is shown in Table 2. Except C18:1, no differences were found in saturated fatty acid, monounsaturated fatty acid, and n-6 polyunsaturated fatty acid compositions between olive oil control, and HMLE groups while none of these fatty acids varied between olive oil control and fish oil control groups. However, n-3 polyunsaturated fatty acids including C18:3n-3, C20:5n-3 (EPA), C22:6n-3 (DHA), and total n-3increased in both fish oil and HMLE groups when compared with the olive oil group.

### 3.2. Effects of HMLE on Intestinal Morphology and Activity of DAO and MPO

**Figure 3:**
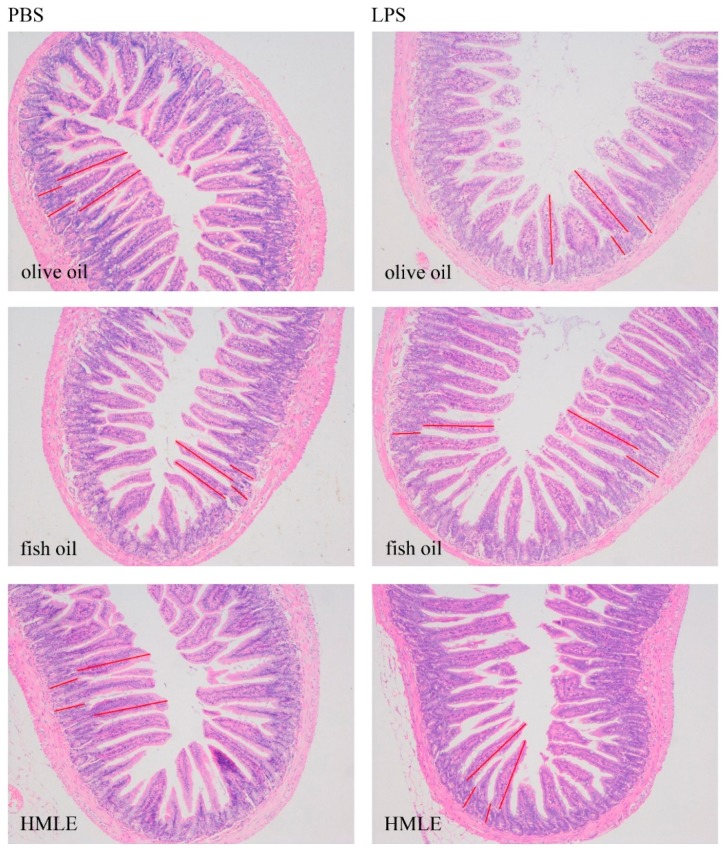
Histological evaluation for the villus height and crypt depth (100×).

Morphology changes in the ileum of mice with LPS-induced intestinal injury are shown in Figure 3. There was no interaction between gavage treatment and LPS observed for the villus height and crypt depth in ileum (Table 3). Compared with mice receiving olive oil and fish oil, mice administered with HMLE had a higher villus height in ileum (*p* for gavage treatment < 0.001). Mice challenged with LPS had a lower villus height in ileum than those challenged with PBS (*p* for LPS challenge < 0.001). Neither gavage treatment nor the LPS challenge had an effect on crypt depth.

Mice challenged with LPS had higher colonic MPO activity but lower DAO activity (both *p* for LPS challenge < 0.001) (Table 3). Significant interaction between gavage treatment and LPS was observed for both colonic MPO (*p* for interaction < 0.001) and DAO (*p* for interaction = 0.05) activity. In comparison with olive oil, the administration of fish oil or HMLE lowered colonic MPO activity and increased DAO activity among LPS-challenged mice while MPO and DAO activity did not differ among PBS-treated mice. It is noteworthy that HMLE lowered MPO activity more than fish oil among LPS-challenged mice.

### 3.3. Effects of HMLE on Colonic Cytokines Secretion

Relative to mice injected with PBS, mice challenged with LPS had higher colonic cytokine concentrations including IL-1β, IL-6, IL-10, and TNF-α (all *p* for LPS challenge < 0.001) (Table 4). A trend for interaction between gavage treatment and LPS was observed for colonic IL-1β (*p* for interaction = 0.06) and IL-6 (*p* for interaction = 0.06) secretion in which mice administered with fish oil or HMLE had lower colonic IL-1β and IL-6 concentrations among LPS-treated mice while IL-1β and IL-6 did not vary among PBS-treated mice. There was a significant interaction between gavage treatment and LPS observed for colonic IL-10 (*p* for interaction < 0.001) and TNF-α (*p* for interaction = 0.04) concentration (Table 4). Similarly, IL-10 and TNF-α concentration did not differ among PBS-treated mice, but mice treated with fish oil or HMLE had a higher concentration of IL-10 and a lower concentration of TNF-α than those treated with olive oil among the LPS-challenged mice.

### 3.4. Effects of HMLE on Colonic mRNA Expression

For colonic mRNA expression levels of tight junction proteins, there was no interaction between gavage treatment and LPS observed for occludin and claudin-1 (Table 5). Treatment with fish oil or HMLE increased mRNA expression levels of these two tight junction proteins (both *p* for gavage treatment < 0.001) relative to the olive oil. Mice challenged with LPS had lower mRNA expression levels of occuldin and claudin-1 (both *p* for LPS challenge < 0.001) in comparison with those treated with PBS. 

Compared with PBS-treated mice, LPS-challenged mice had higher mRNA expression of IL-1β, IL-6, IL-10, and TNF-α (all *p* for LPS challenge < 0.001) (Table 5). Significant interactions between gavage treatment and LPS was observed for colonic mRNA expression of cytokines IL-1β (*p* for interaction = 0.001), IL-6 (*p* for interaction = 0.002), IL-10 (*p* for interaction < 0.001), and TNF-α (*p* for interaction < 0.001). Treatment with fish oil or HMLE was associated with reduced mRNA expression levels of IL-1β, IL-6, and TNF-α and increased levels of IL-10. These effects were larger among LPS-challenged mice than among PBS-treated mice. Notably, the lowering effect of fish oil on the mRNA level of TNF-α was better than that of HMLE among LPS-challenged mice.

Among four key molecules in the TLR-4 signaling pathway, mice challenged with LPS had a higher mRNA abundance of colonic TLR-4 (*p* for LPS challenge < 0.001) and MyD88 (*p* for LPS challenge < 0.001) compared with mice injected with PBS (Table 5). There was a significant interaction between gavage treatment and LPS observed for TLR-4 (*p* for interaction < 0.001). A trend for interaction between gavage treatment and LPS was found for MyD88 (*p* for interaction = 0.07) so that the responses of these variables were lower in mice receiving fish oil or HMLE when compared with those receiving olive oil. These effects were larger among LPS-challenged mice compared with those in the PBS-treated mice. No interaction between gavage treatment and LPS was observed for mRNA expressions of IRAK4 and TRAF6. Neither gavage treatment nor the LPS challenge had an effect on these two genes.

## 4. Discussion

**Figure 4:**
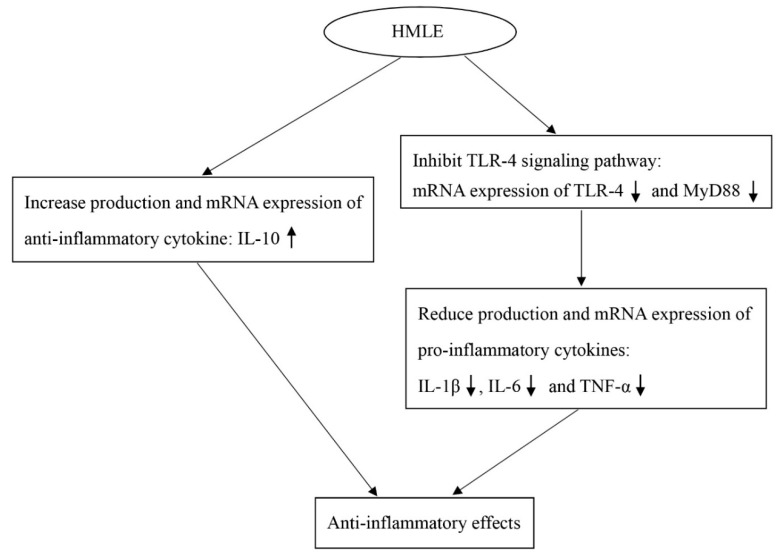
The potential mechanism of a protective effect of HMLE on intestinal morphology and barrier function after an injury induced by LPS using a mouse model (this model could also be applied to fish oils rich in n-3 long chain n-3 PUFA based on the current findings).

It is possible that, due to the high content of n-3 PUFA such as EPA and DHA, HMLE has been shown to exert beneficial effects on arthritis and ulcerative colitis in animal models and in clinical trials [6,13,14,15]. On the basis of this, we examined the potential protective effect of HMLE on intestinal morphology and barrier function after an injury was induced by LPS with the use of a mouse model (Figure 4). In our study, an erythrocyte phospholipid fatty acid profile analysis demonstrated that, like fish oil, administration of HMLE could result in the enrichment of EPA, DHA, and total n-3 PUFA in the mice.

The villus height and crypt depth can be regarded as a criterion to reflect gross intestinal morphology [23]. In the present study, LPS-challenged mice had a lower ileac villus height, which indicates that LPS could cause acute intestinal mucosa damage. In agreement with the previous study [21], our data showed that, independent of the LPS challenge, fish oil could improve intestinal morphology by maintaining the ileac villus height relative to the olive oil. HMLE tended to perform better than fish oil due to having the highest villus height. In response to the LPS-induced inflammation, neutrophils are sequestered from the blood and infiltration will take place in the surrounding tissue [28]. This was substantiated by our results that colonic MPO activity, which is an indicator of the degree of neutrophil infiltration [29], was increased after the LPS challenge. However, the level of colonic MPO activity was dramatically decreased in both fish oil and HMLE groups among LPS-challenged mice, which suggests colonic MPO activity has an anti-inflammatory effect. Similarly, the effect of HMLE was significantly better than the effect of fish oil with lower MPO activity.

DAO is an enzyme deaminating histamine and polyamines and its highest activity is found in the intestinal mucosa in most mammalian species [30]. The activity of DAO in intestinal mucosa decreases when these cells are injured [31]. Therefore, it is a relatively stable marker of intestinal mucosa integrity and barrier function. Our study demonstrated that, compared with olive oil, both fish oil and HMLE increased DAO activity. These results indicate that, like fish oil, HMLE could alleviate the injury of the intestinal barrier function induced by an LPS injection. As for the intestinal barrier function, tight junctions between epithelial cells are the major components [32]. It has been reported that tight junctions are composed of 40 different proteins in which occludin and claudins are thought of as the major integral membrane proteins [33]. Fish oil has been shown to be able to prevent an LPS-induced decrease in occludin and claudin-1 expression in pig models [21]. In the current study, HMLE also tended to enhance the colonic mRNA expression level of occludin and claudin-1 independent of the LPS challenge. HMLE may partially improve the intestinal barrier function by enhancing the mRNA expression of intestinal tight junction proteins.

It is increasingly recognized that cytokines also participate in regulating the intestinal tight junction barrier. Pro-inflammatory cytokines including IL-1β, IL-6, and TNF-α could cause an increase in the permeability of tight junctions [34]. Such effects appear to be an essential mechanism leading to intestinal inflammation. In our study, the LPS challenge significantly increased colonic IL-1β, IL-6, and TNF-α levels while fish oil and HMLE treatments were associated with preventing such increases among LPS-challenged mice. IL-10 is considered an important anti-inflammatory cytokine secreted by T-cells and it seems to be protective against the tight junction barrier disturbance [35]. Our data showed that the colonic IL-10 level was increased after the LPS challenge. This might be due to the nature of the acute study design. It is possible that IL-10 concentration might increase due to the inflammation caused by LPS in the early period, but will decrease in the long run, which was observed in a mouse model where colonic IL-10 concentration was decreased after chronic inflammation [15]. Nevertheless, in the present study, the colonic level of IL-10 was further increased in mice receiving fish oil and HMLE compared with those receiving olive oil. Consistent with attenuated pro-inflammatory cytokine levels and an improved anti-inflammatory cytokine level by fish oil and HMLE administration, fish oil and HMLE decreased the IL-1β, IL-6, and TNF-α gene expression and increased IL-10 relative to olive oil, which suggests that this might be one of the mechanisms of how HMLE prevents the deleterious effects of intestinal injury induced by the LPS challenge in mice.

The transmembrane TLRs are a family of pattern-recognition receptors that play a critical role in recognizing microbial pathogens and modulating the innate immune systems [36]. Among this family, TLR-4 is the most-extensively-studied member. When activated by endotoxin or LPS from Gram-negative bacteria, TLR-4 recruits adaptor proteins such as MyD88 and delivers a signal, which is passed forward by a series of IRAK and TRAF6. This process activates NF-κB, which transduces the signal to the nucleus and results in activation of the inflammatory genes such as pro-inflammatory cytokines [37]. TLR-4 has been reported to be expressed at low levels in basal conditions in vivo but it is upregulated in inflammatory bowel disease [38]. In addition, the effect of LPS was largely inhibited after TLR-4 or MyD88 knockdown and completely blunted in TLR-4 knockout mice [39]. Therefore, the TLR-4 signaling pathway may play a critical role in intestinal inflammation and defense. In the present study, treatment with fish oil and HMLE significantly inhibited the increased expression of TLR-4 and MyD88 in the colon tissue of LPS-challenged mice. Considering the decreased mRNA expression of pro-inflammatory cytokines including IL-1β, IL-6, and TNF-α, it is possible that the protective effects of HMLE administration on the intestinal barrier function were closely related to reducing the expression of intestinal pro-inflammatory cytokines by inhibiting the TLR-4 signaling pathway.

In our study, administration of HMLE significantly improved erythrocyte n-3 PUFA levels in mice and HMLE exhibited comparable or, in some instances, even better protective effects than fish oil on intestinal barrier function during an LPS-induced injury such as restoration of the ileac villus height and inhibition of increased colonic MPO activity. Therefore, similar to fish oil, the n-3 PUFAs in HMLE might be responsible for the protective effects. Some researchers have studied the active components in another marine mussel lipid extract reported to have anti-inflammatory effects—GMLE. They identified some novel anti-inflammatory n-3 PUFAs such as 5,9,12,15-octadecatetraenoic acid, 5,9,12,16-nonadecatetraenoic acid, 7,11,14,17-eicosatetraenoic acid, and 5,9,12,15,18-heneicosapentaenoic acid other than EPA and DHA [40]. However, the protective effects of HMLE could not be solely ascribed to n-3 PUFAs since HMLE was more effective in the previously mentioned aspects than in fish oil. However, the n-3 PUFAs content in HMLE is lower than in the fish oil (124.2 mg/kg vs. 208.7 mg/kg). Likewise, a previous study indicated that GMLE also contained much lower content of n-3 PUFA than that of tuna oil but exhibited much higher anti-inflammatory activity than tuna oil [41]. One study on GMLE reported novel active compounds called furan fatty acids, which do not belong to n-3 PUFAs, but had a higher anti-inflammatory activity than EPA [42,43]. As for HMLE, further studies would be needed to identify new potential active compounds and investigate whether the protective effects could be attributed to any single lipid class, a single fatty acid, or the summative effect of different lipid classes and combinations of different fatty acids.

## 5. Conclusions

In summary, like fish oil, HMLE exerts beneficial effects in improving intestinal integrity after the LPS challenge in mice. It is possible that this protective effect on the intestine might be due in part to regulation of the production and expression of both pro-inflammatory and anti-inflammatory cytokines as well as the downregulation of the TLR-4 signaling pathways.

## Figures and Tables

**Table 1:** Fatty acid composition of olive oil, fish oil, and HMLE.

Fatty Acid	Olive Oil	Fish Oil	HMLE
C14:0	ND	4.37 ± 0.23	2.71 ± 0.09
C15:0	ND	0.27 ± 0.07	0.81 ± 0.06
C16:0	10.79 ± 0.46	8.84 ± 0.37	24.83 ± 0.87
C17:0	ND	0.98 ± 0.08	2.24 ± 0.09
C18:0	3.23 ± 0.16	1.43 ± 0.12	6.31 ± 0.13
C20:0	0.43 ± 0.08	0.12 ± 0.04	ND
Total SFA	14.06 ± 0.98	16.01 ± 1.07	36.90 ± 0.81
C16:1	0.82 ± 0.09	6.31 ± 0.43	7.28 ± 0.11
C17:1	ND	1.79 ± 0.16	3.10 ± 0.07
C18:1	77.27 ± 2.84	6.67 ± 0.54	4.55 ± 0.18
C20:1	ND	0.53 ± 0.03	5.81 ± 0.17
Total MUFA	78.08 ± 2.88	15.30 ± 1.13	20.72 ± 0.67
C18:2n-6	6.38 ± 0.11	0.99 ± 0.07	2.21 ± 0.09
C20:2n-6	ND	0.07 ± 0.02	0.52 ± 0.05
C20:3n-6	ND	0.16 ± 0.05	0.45 ± 0.04
C20:4n-6	ND	1.29 ± 0.34	2.83 ± 0.12
Total n-6 PUFA	6.38 ± 0.11	2.51 ± 0.37	6.01 ± 0.21
C18:3n-3	0.58 ± 0.08	1.01 ± 0.08	1.48 ± 0.06
C20:5n-3	ND	36.51 ± 3.27	13.06 ± 0.64
C22:5n-3 (EPA)	ND	ND	0.60 ± 0.05
C22:6n-3 (DHA)	ND	23.93 ± 2.78	18.15 ± 0.97
Total n-3 PUFA	0.58 ± 0.08	61.45 ± 4.95	33.29 ± 0.96

ND, not detectable; SFA, saturated fatty acid; MUFA, monounsaturated fatty acid; PUFA, polyunsaturated fatty acid; EPA, eicosapentaenoic acid; DHA, docosahexaenoic acid. Data are represented as the mean ± SD.

**Table 2:** Effects of different gavage treatments on mice erythrocyte phospholipid fatty acid composition.

	Olive Oil (*n* = 12)	Fish Oil (*n* = 12)	HMLE (*n* = 12)
16:0	27.16 ± 1.35	27.41 ± 0.16	27.99 ± 4.69
18:0	10.25 ± 0.89	10.30 ± 0.72	9.07 ± 1.36
20:0	0.20 ± 0.03	0.20 ± 0.04	0.20 ± 0.06
22:0	0.58 ± 0.20	0.88 ± 0.26	0.77 ± 0.22
24:0	1.80 ± 0.38	2.01 ± 0.42	2.17 ± 0.37
Total SFA	39.99 ± 1.13	40.80 ± 0.98	40.19 ± 2.05
16:1	0.31 ± 0.05	0.39 ± 0.20	0.34 ± 0.20
18:1	14.85 ± 0.59 ^a^	14.49 ± 0.52 ^ab^	13.62 ± 0.97 ^b^
20:1	0.35 ± 0.10	0.34 ± 0.12	0.30 ± 0.08
22:1	0.15 ± 0.04	0.15 ± 0.04	0.13 ± 0.07
24:1	0.77 ± 0.37	0.78 ± 0.06	0.88 ± 0.51
Total MUFA	16.43 ± 0.77 ^a^	16.16 ± 0.46 ^ab^	15.27 ± 0.97 ^b^
18:2n-6	13.08 ± 0.63	13.27 ± 0.62	13.35 ± 1.00
18:3n-6	0.12 ± 0.05	0.09 ± 0.09	0.08 ± 0.04
20:2n-6	0.35 ± 0.02	0.32 ± 0.03	0.32 ± 0.03
20:3n-6	1.47 ± 0.08	1.41 ± 0.10	1.62 ± 0.22
20:4n-6	17.98 ± 1.46	16.34 ± 1.14	17.74 ± 4.42
22:2n-6	1.71 ± 0.24	1.65 ± 0.23	1.74 ± 0.35
Total n-6 PUFA	34.71 ± 1.53	33.07 ± 1.14	34.86 ± 1.27
18:3n-3	0.11 ± 0.06 ^b^	0.25 ± 0.11 ^a^	0.22 ± 0.08 ^a^
20:5n-3 (EPA)	0.45 ± 0.08 ^b^	0.59 ± 0.11 ^a^	0.54 ± 0.07 ^a^
22:6n-3 (DHA)	8.31 ± 0.32 ^b^	9.21 ± 0.75 ^a^	8.91 ± 0.41 ^a^
Total n-3 PUFA	8.86 ± 0.47 ^b^	10.04 ± 0.87 ^a^	9.67 ± 0.59 ^a^

SFA, saturated fatty acid; MUFA, monounsaturated fatty acid; PUFA, polyunsaturated fatty acid; EPA, eicosapentaenoic acid; DHA, docosahexaenoic acid. Data are represented as the mean ± SD. Values with different letters in each row differ significantly according to one-way ANOVA and the Duncan post-hoc test (*p* < 0.05). The letter ‘a’ represents the highest value.

**Table 3:** Effects of different gavage treatments on ileac villus height and crypt depth and colonic DAO and MPO activity.

	PBS			LPS			*p* Value		
	Olive Oil (*n* = 6)	Fish Oil (*n* = 6)	HMLE (*n* = 6)	Olive Oil (*n* = 6)	Fish Oil (*n* = 6)	HMLE (*n* = 6)	Gavage Treatment	LPS Challenge	Interaction
Ileac villus height (µm)	256.4 ± 15.3 ^b^	255.3 ± 18.5 ^b^	281.4 ± 19.7 ^a^	212.3 ± 22.8 ^c^	222.9 ± 11.9 ^c^	259.2 ± 15.0 ^ab^	<0.001	<0.001	0.40
Ileac crypt depth (µm)	76.9 ± 17.14	76.9 ± 11.9	80.8 ± 20.2	87.3 ± 14.4	85.6 ± 10.2	85.7 ± 16.9	0.94	0.10	0.89
Colonic DAO activity (U/mg protein)	0.21 ± 0.05 ^a^	0.21 ± 0.03 ^a^	0.20 ± 0.03 ^a^	0.08 ± 0.03 ^c^	0.12 ± 0.02 ^b^	0.13 ± 0.02 ^b^	0.36	<0.001	0.05
Colonic MPO activity (U/g tissue)	0.58 ± 0.08 ^d^	0.63 ± 0.09 ^d^	0.57 ± 0.08 ^d^	1.98 ± 0.25 ^a^	1.52 ± 0.09 ^b^	1.30 ± 0.06 ^c^	<0.001	<0.001	<0.001

Data are represented as the mean ± SD. Values with different letters in each row differ significantly according to one-way ANOVA and the Duncan post-hoc test (*p* < 0.05). The letter ‘a’ represents the highest value.

**Table 4:** Effects of different gavage treatments on colonic cytokines secretion.

	PBS			LPS			*p* Value		
	Olive Oil (*n* = 6)	Fish Oil (*n* = 6)	HMLE (*n* = 6)	Olive Oil (*n* = 6)	Fish Oil (*n* = 6)	HMLE (*n* = 6)	Gavage Treatment	LPS Challenge	Interaction
Colonic IL-1β (pg/g tissue)	607.2 ± 70.4 ^c^	589.2 ± 35.5 ^c^	590.9 ± 55.6 ^c^	1034.5 ± 89.1 ^a^	814.9 ± 147.7 ^b^	749.1 ± 96.5 ^bc^	0.03	<0.001	0.06
Colonic IL-6 (pg/g tissue)	378.1 ± 76.3 ^c^	409.2 ± 100.8 ^c^	365.0 ± 76.8 ^c^	932.0 ± 90.4 ^a^	745.7 ± 83.3 ^b^	675.4 ± 88.1 ^b^	0.06	<0.001	0.06
Colonic IL-10 (ng/g tissue)	15.5 ± 2.0 ^c^	14.0 ± 1.8 ^c^	13.1 ± 2.5 ^c^	22.4 ± 1.2 ^b^	29.4 ± 2.6 ^a^	32.4 ± 1.6 ^a^	0.02	<0.001	<0.001
Colonic TNF-α (pg/g tissue)	418.8 ± 77.9 ^c^	403.4 ± 26.6 ^c^	435.9 ± 64.2 ^c^	985.8 ± 46.5 ^a^	848.5 ± 42.6 ^b^	832.9 ± 44.7 ^b^	0.05	<0.001	0.04

Data are represented as the mean ± SD. Values with different letters in each row differ significantly according to one-way ANOVA and the Duncan post-hoc test (*p* < 0.05). The letter ‘a’ represents the highest value.

**Table 5:** Effects of different gavage treatments on colonic mRNA expression.

	PBS			LPS			*p* Value		
	Olive Oil (*n* = 6)	Fish Oil (*n* = 6)	HMLE (*n* = 6)	Olive Oil (*n* = 6)	Fish Oil (*n* = 6)	HMLE (*n* = 6)	Gavage Treatment	LPS Challenge	Interaction
Colonic tight junction proteins									
occludin	1.00 ^b^	1.14 ± 0.07 ^a^	1.14 ± 0.08 ^a^	0.63 ± 0.04 ^c^	0.77 ± 0.07 ^b^	0.82 ± 0.04 ^b^	<0.001	<0.001	0.56
claudin-1	1.00 ^bc^	1.06 ± 0.07 ^a^	1.11 ± 0.08 ^ab^	0.80 ± 0.03 ^d^	0.95 ± 0.07 ^c^	0.98 ± 0.09 ^c^	<0.001	<0.001	0.21
Colonic cytokines									
IL-1β	1.00 ^c^	0.77 ± 0.04 ^d^	0.85 ± 0.04 ^d^	1.71 ± 0.18 ^a^	1.15 ± 0.06 ^b^	1.12 ± 0.32 ^bc^	<0.001	<0.001	0.001
IL-6	1.00 ^c^	0.94 ± 0.03 ^c^	0.94 ± 0.02 ^c^	2.73 ± 0.21 ^a^	1.90 ± 0.10 ^b^	1.87 ± 0.31 ^b^	0.001	<0.001	0.002
IL-10	1.00 ^c^	1.05 ± 0.03 ^c^	1.02 ± 0.04 ^c^	1.47 ± 0.21 ^b^	2.60 ± 0.10 ^a^	2.60 ± 0.20 ^a^	<0.001	<0.001	<0.001
TNF-α	1.00 ^d^	0.70 ± 0.05 ^d^	0.79 ± 0.03 ^d^	7.71 ± 0.54 ^a^	2.43 ± 0.23 ^c^	4.34 ± 0.37 ^b^	<0.001	<0.001	<0.001
Key molecules in the TLR-4 signaling pathway									
TLR-4	1.00 ^c^	0.91 ± 0.04 ^d^	0.91 ± 0.03 ^d^	1.76 ± 0.07 ^a^	1.22 ± 0.04 ^b^	1.23 ± 0.02 ^b^	<0.001	<0.001	<0.001
MyD88	1.00 ^c^	0.97 ± 0.02 ^c^	0.94 ± 0.03 ^c^	3.86 ± 0.37 ^a^	3.14 ± 0.36 ^b^	3.16 ± 0.38 ^b^	0.04	<0.001	0.07
IRAK4	1.00	0.98 ± 0.05	0.97 ± 0.06	1.14 ± 0.19	1.01 ± 0.05	1.04 ± 0.05	0.29	0.14	0.40
TRAF6	1.00	0.97 ± 0.06	0.96 ± 0.07	1.07 ± 0.09	1.04 ± 0.09	0.99 ± 0.05	0.30	0.10	0.82

Data are represented as the mean ± SD. Values with different letters in each row differ significantly according to one-way ANOVA and the Duncan post-hoc test (*p* < 0.05). The letter ‘a’ represents the highest value.
